# Assessment of Adverse Events in Protocols, Clinical Study Reports, and Published Papers of Trials of Orlistat: A Document Analysis

**DOI:** 10.1371/journal.pmed.1002101

**Published:** 2016-08-16

**Authors:** Jeppe Bennekou Schroll, Elisabeth I. Penninga, Peter C. Gøtzsche

**Affiliations:** Nordic Cochrane Centre, Rigshospitalet, Copenhagen, Denmark; Georgetown University Medical Center, UNITED STATES

## Abstract

**Background:**

Little is known about how adverse events are summarised and reported in trials, as detailed information is usually considered confidential. We have acquired clinical study reports (CSRs) from the European Medicines Agency through the Freedom of Information Act. The CSRs describe the results of studies conducted as part of the application for marketing authorisation for the slimming pill orlistat. The purpose of this study was to study how adverse events were summarised and reported in study protocols, CSRs, and published papers of orlistat trials.

**Methods and Findings:**

We received the CSRs from seven randomised placebo controlled orlistat trials (4,225 participants) submitted by Roche. The CSRs consisted of 8,716 pages and included protocols. Two researchers independently extracted data on adverse events from protocols and CSRs. Corresponding published papers were identified on PubMed and adverse event data were extracted from this source as well. All three sources were compared. Individual adverse events from one trial were summed and compared to the totals in the summary report.

None of the protocols or CSRs contained instructions for investigators on how to question participants about adverse events. In CSRs, gastrointestinal adverse events were only coded if the participant reported that they were “bothersome,” a condition that was not specified in the protocol for two of the trials. Serious adverse events were assessed for relationship to the drug by the sponsor, and all adverse events were coded by the sponsor using a glossary that could be updated by the sponsor. The criteria for withdrawal due to adverse events were in one case related to efficacy (high fasting glucose led to withdrawal), which meant that one trial had more withdrawals due to adverse events in the placebo group. Finally, only between 3% and 33% of the total number of investigator-reported adverse events from the trials were reported in the publications because of post hoc filters, though six of seven papers stated that “all adverse events were recorded.”

For one trial, we identified an additional 1,318 adverse events that were not listed or mentioned in the CSR itself but could be identified through manually counting individual adverse events reported in an appendix. We discovered that the majority of patients had multiple episodes of the same adverse event that were only counted once, though this was not described in the CSRs. We also discovered that participants treated with orlistat experienced twice as many days with adverse events as participants treated with placebo (22.7 d versus 14.9 d, *p*-value < 0.0001, Student’s *t* test). Furthermore, compared with the placebo group, adverse events in the orlistat group were more severe. None of this was stated in the CSR or in the published paper.

Our analysis was restricted to one drug tested in the mid-1990s; our results might therefore not be applicable for newer drugs.

**Conclusions:**

In the orlistat trials, we identified important disparities in the reporting of adverse events between protocols, clinical study reports, and published papers. Reports of these trials seemed to have systematically understated adverse events. Based on these findings, systematic reviews of drugs might be improved by including protocols and CSRs in addition to published articles.

## Abstract 2

In a document analysis, Jeppe B. Schroll and colleagues seek insights into potential bias and underreporting in documentation of adverse events from the mid-1990s clinical trials of the anti-obesity drug orlistat.

## Author Summary

**Why Was This Study Done?:**

- Most drugs have adverse effects, or harms, that may become evident in clinical trials.
- Pharmaceutical companies seeking to market a new drug must report adverse effects observed in trial participants in the Clinical Study Reports (CSRs), which they provide to regulatory authorities.
- Additionally, investigators may report harms in published reports of their trials.
- We sought to understand the accuracy, and potential bias, in harms reporting for trials of orlistat, a slimming drug from Roche approved in Europe in 1998 and still marketed in Europe today.

**What Did The Researchers Do And Find?:**

- Using a Freedom of Information Act request to the European Medicines Agency (EMA), we obtained CSRs describing seven clinical trials of orlistat. We studied protocol instructions to investigators for reporting harms, the actual reporting of harms in individual CSR records versus summaries, and the final reporting of harms in published papers.
- We found that protocol instructions to trial investigators had the potential to dilute the appearance of drug-associated harms.
- Between 3% and 33% of the total adverse effects from CSR summaries were described in published papers.
- In one trial, we counted adverse events individually and found that both the number of adverse effects and the number of days with adverse effects in participants taking the drug were understated in the corresponding publication.

**What Do These Findings Mean?:**

- The reporting of trials of orlistat in the 1990s understated harms in the summarised results submitted to the EMA for drug approval and in the published papers.
- Based on the characteristics of harms observed and reported in these trials, we suggest that reports of harms include duration of adverse effects.
- We also suggest that systematic reviews of drugs might be improved by including protocols and CSRs in addition to published articles.

## Introduction

Randomised trials generally underreport harms, which according to Consolidated Standards of Reporting Trials (CONSORT) is the totality of adverse events [1]. In 14% of 185 randomised trials published in major medical journals in 1997, adverse reactions were not mentioned at all, and in 32% they were not shown for each arm, or general statements were used [2]. Only 16% of the trial reports described how adverse events were identified [2], which is also problematic because the way the investigator obtains information impacts greatly on the number [3] and reported characteristics of the events [1]. Another survey found that only 18% of all paediatric randomised trials published between 2006 and 2009 reported harms adequately according to the CONSORT guidelines [4].

Industry-sponsored trials are more likely than other trials to conclude that a drug is safe [5]. A similar bias exists in industry-supported reviews of drugs, which are less transparent, have fewer reservations about methodological limitations of the included trials, and have more favourable conclusions than Cochrane reviews of the same drugs [6].

Selective reporting of harms can have disastrous consequences. Rofecoxib, a selective cox-2 inhibitor, was withdrawn from the market in 2004 due to cardiac adverse events [7]. A study published in 2000 by Merck could have revealed the risk, but due to a nondisclosed cut-off date, not all events were included [8,9]. Pfizer stated that celecoxib does not cause heart attacks at a Federal Drug Administration (FDA) hearing in 2005, despite having evidence to suggest the contrary [10]. In 2009, they called the evidence “inconclusive” in information given to patients invited to participate in a clinical trial [11]. It is estimated that both drugs have caused many deaths due to adverse events [12].

Many steps, decisions, and assumptions precede the reporting or omission of an adverse event. Lack of recorded details has been identified as a problem [13]. Adverse events are coded by the sponsor, which is a potentially bias-prone process. Little is known about whether this process is blinded. In a recent review, we found that reliable interobserver studies of coding have not been conducted, and that modern coding systems might have made statistical detection of adverse events more difficult because of splitting similar events into several categories [14].

When a pharmaceutical company applies for marketing authorisation at a drug agency, they submit an application that includes detailed reports about each of the clinical trials also known as clinical study reports (CSRs). The CSRs are formatted in accordance to a standard developed in 1995 by the International Conference of Harmonisation (ICH) [15].

To better understand these issues of selective reporting, bias, and inadequate recording, we sought to describe how a major pharmaceutical company seeking regulatory approval addressed the issue of collecting and reporting data on harms in its clinical trials. In 2011, we submitted a Freedom of Information request and obtained the CSRs from placebo controlled trials of orlistat—an anti-obesity drug—submitted to the European Medicines Agency (EMA) by Roche for marketing authorisation [16]. Orlistat was approved by the EMA in 1998 but, along with other slimming drugs, has since encountered regulatory barriers. Nearly all slimming pills (but not orlistat) have been withdrawn from European markets because of harms [17–19]. In 2011, the FDA issued a warning regarding orlistat based on 13 cases of liver failure [20]. The CSRs include trials’ protocols and anonymised individual participant data (see Table 1) with narrative descriptions of adverse events. We have used these unique data to study how adverse events and methods for obtaining them were presented in protocols, CSRs, and published papers. The objective was prespecified in our protocol, but we also planned to explore the issues in more detail.

**Table 1:** Example of individual adverse event listings for one participant in trial NM14336, page 6 [21].

USA13866/0015	Treatment	Severity	Relation to drug	Day of onset	Duration	Drug discontinued
BACK PAIN	Placebo	Mild	Unrelated	17	1	NO
UPPER RESP TRACT INFECTION	Placebo	Moderate	Unrelated	36	1	NO
INFLUENZA SYNDROME	Placebo	Mild	Unrelated	165	3	NO
CARBOHYDRATE METABOLISM DISTUR	Placebo	Mild	Unrelated	176	>1	YES

## Methods

Seven placebo-controlled randomised trials of orlistat were included in the application for marketing authorisation. In total, the CSRs consisted of 8,716 pages and included 4,225 participants. The first couple of pages in the CSRs were a brief synopsis followed by module 1 and 2. Module 1 contained the “core report,” which consisted of around 100 pages and was structured by these sections: methods, results and discussion, and conclusion. The module also contained appendices, which consisted of several hundred pages. Selected appendices were an overview of adverse events by organ system, full anonymised individual participant data of adverse events including the information described in Table 1, and detailed narrative descriptions of serious adverse events and events leading to withdrawal from the study. Module 2 contained the study protocol, a blank case report form, a table comparing investigator adverse event terms with the chosen term from the dictionary, bioanalytical report, an investigator list, and a randomisation list. All data were unredacted. We did not receive module 3, 4, and 5 from the EMA, and we determined that information in modules 1 and 2 was sufficient for our analysis. According to a table of contents these modules included participant listings of efficacy data, adverse event listings by organ system, and a statistical report.

For the protocols, two investigators (EIP and JBS) independently extracted names of authors, withdrawal criteria, coding strategies, and information about how adverse events were planned to be recorded and summarised. We also extracted strategies for handling vitamin deficiency (as orlistat decreases absorption of fat from the gut, it might affect the absorption of fat-soluble vitamins) and measures of quality of life (which can potentially reflect harms).

For the core report of the CSRs, the same investigators noted identifiers such as investigator names, start and end dates, treatment duration and countries, and extracted the following data: from the synopsis, all information about investigator-reported adverse events; from the methods section, information about withdrawals, harms, and quality of life; from the results section, the overview of the adverse events section, number of participants, mean age, mean BMI, gender, participants withdrawn, adverse events, serious adverse events, gastrointestinal adverse events, deaths, quality of life scores, liver function tests, increased heart rate, and number of patients with gallbladder diseases and low vitamin levels; and from the discussion section and the conclusion, all text describing adverse events.

We searched PubMed with “orlistat or Xenical” to find the corresponding publications. The search returned 1,433 items, which were screened by one author. We included studies describing investigator-reported adverse events from each of the seven trials included in the application for marketing authorisation. Based on the abstract, we downloaded 35 articles as full text for further evaluation. We identified nine papers that described the seven trials individually. Each trial had a detailed primary publication that summarised investigator-reported adverse events [22–28] and was included in our study. The two additional publications did not contribute additional data about adverse events [29,30] and were excluded. An additional seven papers with pooled estimates from the trials did not contain investigator-reported adverse events and were excluded [31–37]. One paper explored abnormal liver function test [37], which we had extracted from the CSRs. We had planned to examine how investigator-reported liver safety of orlistat was reported in publications, but since none of the trial reports had investigator-reported adverse events related to the liver, we excluded the study. The remaining studies were excluded because they did not describe one of the seven trials from the application. We extracted information about adverse events from the seven primary publications.

We converted all individual participant adverse event listings from the CSR for one trial (trial 7) by using text recognition software (ABBYY FineReader 10) and transferred the data to Excel. Trial 7 was chosen as an example because it was the newest and also one of the smallest, and it had a relatively simple design.

We studied how adverse events were categorised, recorded, and analysed by comparing protocols, CSR core reports, and publications. We also looked for signals within the CSRs of elevated liver function tests and vitamin deficiency but did not compare this or other abnormal laboratory values to publications, as they are not traditionally considered adverse events and there is less guidance on how they should be reported. Adverse events reported in the CSRs were compared with corresponding publications, and in trial 7 we checked whether adverse events were summarised consistently with the individual adverse events listed in an appendix.

## Results

### Trial Design

The protocols described seven phase III randomised trials, all with a placebo arm, and with orlistat given as 30 mg, 60 mg, or 120 mg three times a day (Table 2). The duration of the studies was 52 to 104 wk. Trial 2 re-randomised the participants to either placebo or orlistat after 52 wk of treatment and trial 5 changed the dose after 52 wk for half the participants. Participants and treating physicians were blinded to the treatment, but whether the coders of adverse events were blinded was not mentioned in any of the documents.

**Table 2:** Overview of trials included in the present study.

Trial ID	Start/end date	Lead-in* /duration	Countries	Patients	Treatment arms (1 y/2 y)	*n*, participants	Threshold for reporting GI adverse events**
1—BM14119B	June 1992–August 1994	4 wk/ 52 wk	United Kingdom	BMI 30–43	120 mg TID	114	3%
					Placebo	114	
2—BM14119C	May 1992–October 1995	4 wk/ 104 wk	Austria, Denmark, Finland, France, Germany, Netherlands, Sweden and Switzerland	BMI 30–43	120 mg TID/120 mg TID	135	1%
					120 mg TID/placebo	138	
					placebo/120 mg TID	127	
					placebo/placebo	126	
					3—BM14149	May 1993–February 1996		4 wk/ 104 wk	Austria, Finland, France, Germany, Netherlands, Sweden and Switzerland	BMI 28–43	60 mg TID	242	1%
120 mg TID	244												
Placebo	243												
4—NM14161	February 1993–December 1995	4 wk/ 104 wk	United States, primary care	BMI 30–43	60 mg TID	214	1%
					120 mg TID	214	
					Placebo	214	
5—NM14185	October 1992–October 1995	4 wk/ 104 wk	United States	BMI 31–43	120 mg TID/120 mg TID	153	1%
					120 mg TID/60 mg TID	152	
					120 mg TID/placebo	138	
					placebo/placebo	133	
6—NM14302	May 1993–March 1996	24 wk/ 52 wk	United States	BMI 28–38	30 mg TID	187	3%
					60 mg TID	173	
					120 mg TID	181	
					placebo	188	
7—NM14336	December 1993–January 1996	5 wk/ 52 wk	United States	BMI 28–40 per oral treated Type 2 Diabetics	120 mg TID	163	3%
					placebo	159	

* Lead-in was a period in which both groups got placebo.

** Threshold for reporting GI adverse events in CSRs core report. All data was available in appendices.

TID, three times a day; GI, gastrointestinal.

The trials were conducted between 1992 and 1996 in the United States and Europe. They all had a lead-in period, which mostly lasted for 4–5 wk, when the participants received placebo along with dietary advice. Based on pre-defined criteria some participants were excluded based on their performance in this period. The included participants had a BMI between 28 and 43. Trial 7 only included participants with type II diabetes, whereas the other trials excluded such participants.

### Protocols

All protocols mentioned that vital signs, adverse events, routine laboratory tests, fat-soluble vitamin levels, and ECG should be recorded. All protocols had at least eight withdrawal criteria. Apart from “new smokers,” which was an additional criterion in five protocols, the withdrawal criteria were the same. “Administrative reasons” or “other reasons” were sufficient for withdrawal and were not further specified.

Three protocols (trials 1–3) contained an appendix on how to code adverse changes in defecation patterns; these appendices included guidance and a term list for adverse events, with descriptive definitions for each term (all events were in American English; for consistency, we have used British English). All three protocols read: “In this dictionary the term diarrhoea and constipation has been avoided. In fact, the use of these terms could cause some misunderstandings, as there is no well-accepted definition…” [38]. Instead the following categories were used: “increased defaecation,” “liquid stools,” “soft stools,” “fatty/oily evacuations,” “oily spotting,” “faecal urgency,” “faecal incontinence,” “flatus with discharge,” “decreased defaecation,” “pellets,” and “solid stools.” Even though protocols 4–7 did not contain this appendix, it may have been used because, for all these trials, instances of “diarrhoea” in the CSRs list of adverse events were re-categorised.

The protocols included between 9 and 17 planned visits per participant during the first year, and adverse events were to be recorded at each visit on the case report forms. Only a change from the participants’ pre-treatment condition was to be considered an adverse event, and the protocols provided no guidance on how to question the participants. The investigator was to relate the severity to daily function and also judge the relationship to treatment (two CSRs contained an appendix which offered guidance on this).

For quality of life, six protocols specified that the main outcome was “comparative rates of change” for the subscales “health distress and emotional functioning.” The scales were not specified in any of the protocols; instead, they referred to a questionnaire included in the protocol, which was a 46-item list divided into seven subscales with no information about how the scores from the subscales were to be combined. Secondary quality of life outcomes were simply described as “a variety of scales.”

Fat-soluble vitamin levels were monitored in a blind fashion and vitamin supplements were prescribed if vitamin levels were below a threshold on two consecutive occasions. One study provided multivitamin tablets for all patients.

The only information on the statistical handling of adverse events was that the treatment groups would be compared using “descriptive statistics.”

### Clinical Study Reports: Methods

As in the protocols, it was not specified how the participants had been questioned about adverse events. Coding guidelines for gastrointestinal adverse event were also provided in the CSRs but in contrast to the protocol (trial 1 and 2) some of the terms were marked with an asterisk. The terms without an asterisk should only be considered adverse events when “described as bothersome by the patient” and these included “fatty/oily stool,” “liquid stools” (which term the protocol suggested to be used instead of diarrhoea), “increased defaecation,” “stools soft,” “decreased defaecation,” and “pellets.” “Bothersome” was not a requirement for adverse events outside the gastrointestinal category and was not mentioned in the protocols except for trial 3. Furthermore, according to the narrative descriptions in an appendix to the core report, serious adverse events had been assessed for relationship to the drug by the sponsor, although this was not prespecified in the protocol.

The adverse events were coded according to a Ciba-Geigy modified WHO glossary, which could be updated by the sponsor. For each adverse event described by the investigator, the sponsor would assign a preferred term from the dictionary: “For classification purposes, preferred terms were assigned by the sponsor to the original terms for concomitant medications, diseases, and adverse events entered on the case report form” (trial 2, 3, and 6).

In all CSRs, the methods section described that adverse events would be presented as listings and summary tables by body system, intensity, and relation to drug. For gastrointestinal problems, however, only events more frequent than 1% (four trials) or 3% (three trials) would be summarised.

In all CSRs, the methods section noted that the “primary measure” for quality of life was “overweight distress,” “depression,” and “satisfaction with treatment.” We could not find any explanation, in the CSRs or in the amendments, why the primary outcome for quality of life from the protocol had been changed from “health distress and emotional functioning.”

### Clinical Study Reports: Results

All CSRs narratively acknowledged that there were many adverse events but also noted that the differences between placebo and active treatment were small, and two CSRs (trial 2 and 3) noted that most adverse events were considered unrelated to the drug by the investigator. Only one CSR (trial 6) mentioned the total number of participants with one or more adverse events in the results section of the core report. None of the core reports mentioned the total number of events for which the difference between placebo and orlistat group was considerably higher, but the information was available in tables.

The increased number of gastrointestinal adverse events observed in the orlistat group was mentioned but it was claimed that this was due to the pharmacological effect of the drug. It was noted that the numbers of gastrointestinal adverse events per participant were often few (1 to 2), and in the core report, there was no information on their duration. All CSRs contained an appendix in module 2 that documented how original investigator terms were coded. In six out of the seven CSRs, investigators used the term “diarrhoea” but in all cases it was re-categorised as “liquid stools” (page 150 [39]). This was not mentioned in the protocol.

Low vitamin levels were common, particularly for vitamin D, for which low levels were found in 19% of the participants receiving orlistat in the largest trial. Low vitamin E and beta carotene levels were the second most common deficiency, which led to additional substitution but rarely withdrawal from the study. The proportion of participants with affected liver function tests was comparable between the two groups. High alanine transaminase and aspartate transaminase were found in 6.2% and 2.1% of the patients in the orlistat group, respectively, and in 6.3% and 2.3% of the placebo patients, respectively. There was no consistent pattern in heart rate changes or in participants with new gallbladder disease.

More participants in the orlistat treatment group were withdrawn due to adverse events (8.1% versus 4.6%, χ^2^-test *p* < 0.0001) whereas more participants in the placebo group were withdrawn for “any reason” (28.7% versus 22.0%, χ^2^-test *p* < 0.0001). In trial 2, more participants “lost to follow-up” were withdrawn from the placebo group (22 versus 12) and also more participants who “did not cooperate” (26 versus 13). In trial 4, more placebo participants were excluded due to “administrative reasons” (29 versus 10 during the first year).

### Publications

A brief summary of the papers describing the seven clinical trials are listed in Table 3 [22–28]. There were between 71 and 270 times as many pages in the CSRs as in the corresponding publications. Even though the number of unpublished adverse event is less, the compression factor highlights that a lot of data is being omitted when the study is reported in a publication. Six papers described that “all adverse events were recorded,” (all except trial 5) and one informed that the Ciba-Geigy dictionary was used (trial 3).

**Table 3:** Overview of publications included in the present study.

	Citation	Time between completion and publication	Restriction of published adverse events	Number of adverse event in CSR	Number of adverse events in published paper	Percentage of adverse events published	Compression factor*
Trial 1	Finer 2000 [22]	6 y	Adverse events considered “unrelated” by investigator were censored. Only adverse events more common than 3% published.	661	220	33% orlistat	71
				534	112	21% placebo	
Trial 2	Sjoström 1998 [23]	3 y	Adverse events considered “unrelated” by investigator censored. Only adverse events more common than 5% published.	1,511	483	32% orlistat	254
				1,086	162	15% placebo	
Trial 3	Rossner 2000 [24]	4 y	“Common” adverse events reported.	1,097	164	15% orlistat 60 mg	88
				1,280	208	16% orlistat 120 mg	
				1,087	38	3% placebo	
Trial 4	Hauptman 2000 [25]	5 y	Only gastrointestinal adverse events that were considered possibly or probably related to treatment for which incidence in active arm was twice that of placebo were reported.	1,728	253	15% orlistat 60 mg	173
				1,737	240	14% orlistat 120 mg	
				1,327	35	3% placebo	
Trial 5	Davidson 1999 [26]	4 y	No table. Adverse events more frequent than 5% and more than twice as common in orlistat were reported (only orlistat arm events shown).	1,359	Adverse events not reported for placebo arm.	Calculation not possible.	270
				1,483			
				1,387			
				1,147			
Trial 6	Hill 1999 [27]	3 y	No table. “Some gastrointestinal events occurred in a greater percentage of patients in the orlistat-treated groups” was reported in text but the total number of participants was unclear.	1,138	Adverse events not clearly reported.	Calculation not possible.	128
				1,083			
				1,243			
				894			
Trial 7	Hollander 1998 [28]	2 y	No table. Adverse events more frequent than 5% and more than twice as common in orlistat were reported (only orlistat arm).	1,198**	Adverse events not reported for placebo arm in publication.	Calculation not possible.	113
				930			

* Number of pages in CSRs (only module 1 and 2 were available to us) divided by pages in publication.

** During our study we discovered that the counts for trial 7 were too low. While we did not count events for the remaining six trials, under-reporting may have occurred, as many aspects of adverse event recording were similar.

Five papers (trials 2, 3, 4, 6, and 7) mentioned that a special dictionary was developed for the expected gastrointestinal adverse events, but none described that only “bothersome” adverse events should be recorded and none described that “diarrhoea” was discouraged as a term.

All papers had severe restrictions on which adverse events were reported, and only four papers presented a table summarising adverse events (trial 1–4). Two papers censored all events that had been considered “unrelated” (trials 1 and 2) by the investigator and only reported events occurring in 3% or 5% of participants. One paper censored both “unrelated” and “remotely related” events (trial 4). Three papers reported only adverse events that were twice as frequent in the orlistat group as in the placebo group (trials 4, 5, and 7), and two of those had the additional criterion that only events occurring in at least 5% of the participants would be reported (trials 5 and 7). These two papers only reported the adverse event rate for the orlistat group.

For four trials, we could extract data on the number of adverse events, and between 3% and 33% of those reported in the CRSs were also reported in the publications (see Table 3). However, the true percentage reported is probably lower, as the grand total of adverse events reported in the CSRs was also lower than in the individual participant data (see trial 7 below).

Only trial 3, which had the greatest difference between placebo and orlistat, reported on quality of life, but there were no data in the paper, only *p*-values.

All publications mentioned the impact on vitamin measurements. Most reported number of participants who received additional supplements and some reported mean vitamin levels for the entire population.

Trial 1 grouped the gastrointestinal adverse events into two new main categories: “uncontrolled oily discharge,” which included faecal incontinence, flatus with discharge, and oily spotting, and “loose stools,” which included oily evacuation, fatty/oily stool, liquid stools, and soft stools.

### Trial 7, People with Type II Diabetes

In trial 7 almost all participants experienced one or more adverse events (157 participants [96%] in the orlistat groups and 150 [94%] in the placebo group). When we counted the individual participant adverse events we found a total of 3,446 adverse events (2,008 in the orlistat group and 1,438 in the placebo group). These numbers could not be found in the CSR or in the publication, and more events were missing for orlistat than for placebo: in a summary in an appendix in the CSR, the total adverse event count was 1,198 for the orlistat group (60% of our count) and 930 for placebo group (65% of our count). We discovered that multiple events occurring in the same study participant were only counted once; this was not explained in the CSR. We calculated that each participant had 12.8 adverse events, on average, in the orlistat group and 9.6 in the placebo group, corresponding to 3.2 (95% CI: 1.2–5.2, unpaired *t* test) more adverse events in the orlistat group. This was not mentioned in the report or publication.

The duration of each adverse event was recorded, but was not summarised in the CSR or in the publication. We calculated that the average duration was 22.7 d (95% CI: 20.1–25.2) in the orlistat group and 14.9 d (95% CI: 13.1–16.8) in the placebo group and that the number of days each person was affected by an adverse event was 288 d in the orlistat group and 141 d in the placebo group. Thus, on average, orlistat led to double as many days with adverse events as placebo.

The CSR noted that most adverse events were mild to moderate in intensity. However, we found that the events were more severe in the orlistat group (*p* < 0.001, χ^2^ test, not adjusted for dependent observations), which was not mentioned in the CSR or the publication. The relative risk for having a mild adverse event in the orlistat group compared to the placebo group was 0.93 (95% CI: 0.89–0.96); a moderate event, 1.29 (95% CI: 1.13–1.48); and a severe event, 1.39 (95% CI: 0.75–2.59).

More placebo participants were withdrawn due to adverse events (23 versus 12, *p* = 0.04, χ^2^ test) which is unusual. However, 14 of the 23 withdrawn participants in the placebo group were discontinued due to abnormal fasting glucose, and this was categorised as withdrawal due to adverse events. The protocol stated that fasting glucose above 220 mg/dl would lead to discontinuation, but only 2 of the 14 withdrawals were listed as an adverse event in the detailed list of adverse events for each participant. Orlistat protects against hyperglycemia, and was also an efficacy outcome in the trial. With the used criteria, the number of withdrawals due to adverse events appears to be more common in the placebo group even though the published paper reported withdrawals due to gastrointestinal adverse events as well. Furthermore, a baseline imbalance in HbA1c could perhaps partly explain the difference (HbA1c was 8.05 in the active group and 8.20 in the placebo group, Student’s *t* test, *p* = 0.19). In the first quarter of the trial, 14% of participants on orlistat had a hypoglycaemic episode versus 10% on placebo. In the second quarter, the rates were 12% and 6%, respectively. The CSR referred to an appendix regarding more information, but this was missing. We found 426 hypoglycaemia events in the orlistat group and 300 in the placebo group and an average of 2.7 events per participant in the orlistat group and 2.0 in the placebo group (*p* = 0.10, unpaired *t* test).

## Discussion

We found several non-predefined practices in the CSRs and publication that could potentially have resulted in biased reporting of drug-related harms. We had access to protocols, amendments, and content of information of the documents the EMA had not made available to us, and since the filters were not described, they have likely been introduced post hoc. The analysis plan for harms in the protocol consisted of only four lines of text for each CSR. Some gastrointestinal adverse events were only coded if considered “bothersome,” and “diarrhoea” was split into several categories, which can lead to dilution of signals. Only a fraction of adverse events were reported in publications due to various non-predefined censoring filters. Results sections in the core reports of the CSRs often stated that most of the adverse events were considered unrelated to the drug and that they were generally mild to moderate. The many gastrointestinal adverse events were explained as part of the pharmacological effect of orlistat. In one trial we found 11 more participants withdrawn due to adverse events in the placebo group, but this was caused by a high fasting glucose. Fasting glucose and weight loss are correlated, so it was expected that fasting glucose would be higher in the placebo group. With these 11 participants from the placebo arm categorised as withdrawn due to adverse events, adverse events in the orlistat arm may seem less salient. Duration of adverse events was not analysed, even though it was recorded, and no explanation for this was given. In trial 7, including duration of adverse events in the analysis revealed that each treated participant had almost twice as many days with adverse events. Subscales of quality of life were changed without explanation. One aspect of the study design in itself could be a hindrance for identifying adverse events. All trials had a long lead-in period on placebo, from 4 to 24 wk, in which more than 90% of the participants reported at least one adverse event (“complaints”). Should any of these events reoccur during the trial, they would be censored. Since gastrointestinal complaints are normal in healthy people, this type of censoring might have made it more difficult to detect gastrointestinal adverse events caused by orlistat.

Data from observational studies provide an additional perspective on potential harms from orlistat. Slimming pills are often discontinued by the participants [40]. A Canadian study of 16,968 participants on orlistat showed that after 1 y, only 6% of the participants were still taking the drug, and after 2 y, it was only 2% [40]. This suggests that the participants perceive the balance between harms and benefit as unfavourable.

Even though orlistat was approved in 1998, our findings are still relevant. First of all, many drugs approved in this time period are still being sold in large numbers. Secondly, we cannot be sure that analysis plans have improved. Standardised medical dictionaries are now obligatory [14] and the ICH has a guideline on how CSRs should be reported, but analysis of duration of adverse events is still optional and the standard leaves room for interpretation, and therefore, a risk of bias [15]. More supplemental material is available today, but we still need access to protocols to see how authors have arrived at their summary tables. Recent studies have also questioned the quality of the adverse event information in the case report forms [13].

CSRs contain a lot of additional data [41]. Even though it takes more time to use CSRs rather than publications for systematic reviews and meta-analyses, we believe it is worthwhile, as some of the filters used in the case of orlistat would not have been identified in a publication.

Even though publication bias is well covered in the medical literature, few studies have analysed clinical study reports, which in the future could be a very important source of information. Other studies have found that only a fraction of adverse events were reported in published papers compared to the CSRs [42,43]. In one of the studies, Wieseler et al. had access to CSRs of different interventions, and their results might therefore be more generalisable. In our study, we have tried to highlight potential mechanisms of bias that need to be investigated in a confirming study. Our research emphasises the need for detailed analysis plans for harms data.

### Limitations

Our study was explorative and restricted to one drug tested in the mid-1990s; our results might therefore not be applicable to newer drugs. The lack of reporting of important harms could be the consequence of space restrictions in paper journals and could therefore be less of a problem today when electronic appendices are a possibility. Furthermore, standards for reporting CSRs and publications have been developed since the orlistat trials were reported. The CSRs obtained from the EMA and some of the missing modules contained listings related to harms. Based on the table of contents of the missing modules we do not believe access to this data would change our results.

### Conclusion

The protocols, CSRs, and publications all reported poorly on how adverse events were planned to be collected, summarised, and analysed. Censoring filters were introduced post hoc, and the guidance on how to code adverse events differed between protocols and CSRs and was absent in publications. The duration of the adverse events was not included in any of the analyses conducted by the sponsor even though the difference between orlistat and placebo was large. Clinical study reports, protocols, and individual participant data should be the primary data sources for systematic reviews of drugs.

## Supporting Information

S1 Protocol The protocol for this study. (DOCX)

## Abbreviations

CONSORT: Consolidated Standards of Reporting Trials
CSR: clinical study report
EMA: European Medicines Agency
FDA: Federal Drug Administration
ICH: International Conference of Harmonisation
