# Supplementary material for: Assessment of Adverse Events in Protocols, Clinical Study Reports, and Published Papers of Trials of Orlistat: A Document Analysis
Source: PLoS Med. 2016 Aug 16;13(8):e1002101. doi: 10.1371/journal.pmed.1002101 (PMC4987052; doi:10.1371/journal.pmed.1002101)
Supplement: S1 Protocol — (DOCX) [file pmed.1002101.s001.docx]

**Assessment of harms in clinical trials of the anti-obesity drug orlistat
Jeppe Bennekou Schroll, Elisabeth Penninga, Peter Gøtzsche**

**Introduction**

Approved drugs have again and again been withdrawn from the market because they did too much harm. Randomised trials are often too small and too short to identify adverse events. Furthermore companies often downplay or omit serious adverse events in articles. In this project we will evaluate adverse events for the anti-obesity pill orlistat using internal reports, which until recently have been secret, and investigate other potential problems with the handling of adverse events. Based on our analyses we will suggest better strategies of handling and presenting adverse events.

**Background**

The decision to prescribe a drug is based on the balance between the drug’s benefits and harms. Randomised clinical trials are the most reliable source for both effects, but they tend to focus on benefits and to underreport harms. As an example, 14% of 185 randomised trials from 7 eminent medical journals published in 1997 did not mention adverse reactions at all, and in 32% of the trials, adverse events were not specified for each arm or general statements were used.^1^ Only 16% of the trials described how adverse events were indentified^1^, which is problematic because the way the investigator elicits information about adverse events has great impact on the number^2^ and characteristics of events.^3^

Reporting of harms generally does not live up to accepted standards, neither for trials, nor for systematic reviews. Only 19 of 107 trials (18%) in children reported safety data adequately according to the CONSORT guidelines.^4^ In systematic reviews of harms, the search strategies are generally inadequate and are not reported thoroughly^5^, and although it is standard to assess the risk of bias in trials included in reviews and state sources of funding, both were done in less than half of the meta-analyses of adverse events.^6^

Most drug trials are funded by the pharmaceutical industry, and industry-sponsored trials are more likely than other trials to conclude that a drug is safe.^7^ A similar bias exists in industry-supported reviews of drugs, which are less transparent, have few reservations about methodological limitations of the included trials, and have more favourable conclusions than Cochrane reviews of the same drugs.^8^

Selective reporting of harms can be deliberate and it can have disastrous consequences, as illustrated by the COX-2 inhibitors. Merck concealed cases of myocardial infarction and deaths with rofecoxib, which were missing in reports of the pivotal trials.^9, 10^ Pfizer denied that celecoxib causes heart attacks at a FDA hearing in 2005, despite having unpublished evidence to the contrary^11^, and still called the evidence “inconclusive” in 2009 in information to patients invited to take part in a trial.^12^ It is estimated that 60,000 people worldwide lost their lives as a consequence of late withdrawal of rofecoxib from the market.^13^

Many steps, decisions and assumptions precede the reporting of an adverse event in an article or product summary. If a patient describes an adverse event, the investigator sends a report to the sponsor who codes the description for practical purposes. In an almost completed review article we have established that coding has never been validated and that no reliable interobserver study has been conducted. We also concluded that modern coding systems might have made statistical detection of adverse events more difficult and we suggest that more sensitive techniques should be used.^14^

^15^

We have acquired access to the clinical study reports of all the placebo-controlled trials submitted to the European Medicines Agency (EMA) for marketing authorization for the anti-obesity drug, orlistat, and their corresponding trial protocols.^16^ These documents include individual patient data with narrative descriptions of adverse events. We will use these unique data for an in-depth exploration of the problems related to the reporting of harms in drug trials.

**Objectives**

1. To study how adverse events are coded in the orlistat trials.
2. To study how adverse events are reported for orlistat in clinical study reports, package inserts and published papers.
3. To study how information about adverse events are gathered and to compare how the methods are reported in clinical study reports versus published papers.
4. To compare the withdrawal rates in the randomised clinical studies of orlistat with postmarketing observational studies.

In Europe, all marketed diet pills have been withdrawn except for orlistat. In the 1960’s, the diet pills were taken off the market because of pulmonary hypertension and death.^17^ In the following decades several drugs shared this fate. In 2010, sibutramine was withdrawn due to increased risk of myocardial infarction^18^ leaving European doctors with only one antiobesity drug.^19^ Several of these drugs increases the heart rate, which has been associated with early death.^20^ In 2011, 12 cases of liver failure led the FDA to issue a warning about orlistat.^21^ Because of the withdrawal of many drugs in the class and because adverse events are known to be suppressed in drug trials^10^, it is important that orlistat is investigated by independent researchers.

We have access to 7 study reports of the placebo-controlled randomised trials that were included in the application for marketing authorization of orlistat. The reports total 8,716 pages and include 4,225 patients. They contain full trial protocols, an overview of adverse events by organ system, onset and intensity of adverse events for all patients, and detailed narrative descriptions of serious adverse events and events leading to patient withdrawal from the study.

Our research group has experience in analysing huge clinical study reports due to an ongoing study where we investigate suicidality with antidepressants.

**Project 1 - coding**

A) We will validate the coding in the study reports from EMA by having two physicians assess whether each code is clinically accurate. Each study report has tables that correlate the description of an adverse event used by the investigator with the term chosen by the medical coder. Around 1,500 investigator terms are reported for each of the 7 trials.
B) We will conduct an interobserver study of coding. In total approximately 200 serious adverse events are presented with a narrative in our material. We will blank out the treatment arm, preferred term chosen (medical code), intensity, and relation to study drug determined by the investigator and the sponsor. Two independent observers will read the narrative and, based on that, choose a preferred term, assess intensity (mild, moderate, severe) and determine relation to study drug (unrelated, remote, possible, probable, definite). We will determine the interobserver agreement and compare the assessments with the ones made by the sponsor.

**Project 2 – reporting of harms**

We will use individual patient data listed in the clinical study reports submitted to the EMA for marketing approval and compare these with the harms in summary tables in the same reports, with the harms in published reports, and with the harms listed in the package inserts as approved by the FDA and the EMA. Individual patient data is available as scanned documents. We will therefore use text recognition software and afterwards import data into a database in order to be able to reproduce summary tables. To control the quality of the data transfer we will do random checks. We will investigate whether orlistat affects liver function. Due to low expected prevalence we will use sensitive techniques known from pharmacovigilance that lumps related adverse events together, i.e. jaundice, liver failure, affected liver function test, etc.

Identification of published studies

We will search PubMed, use the existing Cochrane review and ask the manufacture, Roche, about published articles based on the 7 trials included in the application for approval.

Data extraction

Two independent researches will extract data from the articles and the study reports and insert them into an Excel spreadsheet. For each treatment arm we will extract the number of adverse events, serious adverse events, withdrawals, deaths, gastrointestinal adverse events, number of patients with elevated liver enzymes, number of patients with vitamin deficiencies, number of patients with increased heart rate and number of patients with gallbladder diseases. Disagreements will be resolved by discussion. From package inserts we will extract serious adverse events, very common adverse events (more than 10%), common adverse events (1-10%) and any affected laboratory values.

**Project 3 – reporting of methods**

Evaluation of the methods used for identifying harms with orlistat. We will compare the methods as described in the trial protocols with those described in the clinical study reports and in published clinical trial reports. Furthermore, we will assess whether the methods used are adequate for identifying the most important harms.

**Project 4 – trials vs. observational studies**

We will conduct a systematic review of cohort studies of orlistat that describes the withdrawal rate after one year or more. This rate will be compared with the rate described in the randomised trials. Withdrawal rates in observational studies have been shown to be very high.^22^ The reasons for withdrawal will be compared, and criterias for withdrawal in the protocols will be examined to elucidate whether they might bias the results in favour of the drug.

**Statistics**

We have access to all controlled placebo trials used for the approval of orlistat. It consists of data from 4,225 patients describing adverse events on 1,053 pages with roughly 30 adverse events per page. We have access to an astonishing 30,000 individual adverse events, which will be more than sufficient to thoroughly investigate how this data is processed in a clinical study report and how it compares with the published papers.

There are 257 narratives of serious adverse events that can be used to determine agreement between different coders. We will use kappa statistics.

**Dissemination**

We will disseminate our findings by publishing the results in major journals, and give oral presentations at relevant conferences. Our research is expected to lead to improvements in the way adverse events are reported in general, potentially benefiting millions of patients. In particular, we are expecting to contribute to determine whether benefits outweigh harms for orlistat. The clinical reports were released from the EMA to protect patients and give them a transparent estimate of harms. We expect to do just that.

Reference List

(1) Loke YK, Derry S. Reporting of adverse drug reactions in randomised controlled trials - a systematic survey. *BMC Clin Pharmacol* 2001;1:3.

(2) Olsen H, Klemetsrud T, Stokke HP, Tretli S, Westheim A. Adverse drug reactions in current antihypertensive therapy: a general practice survey of 2586 patients in Norway. *Blood Press* 1999;8:94-101.

(3) Ioannidis JP, Evans SJ, Gotzsche PC et al. Better reporting of harms in randomized trials: an extension of the CONSORT statement. *Ann Intern Med* 2004;141:781-788.

(4) de Vries TW, van Roon EN. Low quality of reporting adverse drug reactions in paediatric randomised controlled trials. *Arch Dis Child* 2010;95:1023-1026.

(5) Golder S, Loke Y, McIntosh HM. Poor reporting and inadequate searches were apparent in systematic reviews of adverse effects. *J Clin Epidemiol* 2008;61:440-448.

(6) Golder S, Loke Y, McIntosh HM. Room for improvement? A survey of the methods used in systematic reviews of adverse effects. *BMC Med Res Methodol* 2006;6:3.

(7) Golder S, Loke YK. Is there evidence for biased reporting of published adverse effects data in pharmaceutical industry-funded studies? *Br J Clin Pharmacol* 2008;66:767-773.

(8) Jorgensen AW, Hilden J, Gotzsche PC. Cochrane reviews compared with industry supported meta-analyses and other meta-analyses of the same drugs: systematic review. *BMJ* 2006;333:782.

(9) Mukherjee D, Nissen SE, Topol EJ. Risk of cardiovascular events associated with selective COX-2 inhibitors. *JAMA* 2001;286:954-959.

(10) Curfman GD, Morrissey S, Drazen JM. Expression of concern reaffirmed. *N Engl J Med* 2006;354:1193.

(11) Harris G. Pfizer says internal studies show no Celebrex risks. *New York Times* February 5, 2005;sect. Business section.

(12) de AM. Research Ethics . In clear sight. *BMJ* 2009;339:b3443.

(13) Deer B. Vioxx death toll may hit 2,000 in UK. <http://www.timesonline.co.uk/tol/news/uk/article557471.ece> (accesed 26th of august 2011). *The Sunday Times* 2005.

(14) Schroll J, Maund E, Gotzsche PC. Coding adverse events in clinical trials. *(Unpublished)* 2012.

(15) Ioannidis JP, Evans SJ, Gotzsche PC et al. Better reporting of harms in randomized trials: an extension of the CONSORT statement. *Ann Intern Med* 2004;141:781-788.

(16) Gotzsche PC, Jorgensen AW. Opening up data at the European Medicines Agency. *BMJ* 2011;342:d2686.

(17) Curfman GD. Diet pills redux. *N Engl J Med* 1997;337:629-630.

(18) Curfman GD, Morrissey S, Drazen JM. Sibutramine--another flawed diet pill. *N Engl J Med* 2010;363:972-974.

(19) Sayburn A. Withdrawal of sibutramine leaves European doctors with just one obesity drug. *BMJ* 2010;340:c477.

(20) Cooney MT, Vartiainen E, Laatikainen T, Juolevi A, Dudina A, Graham IM. Elevated resting heart rate is an independent risk factor for cardiovascular disease in healthy men and women. *Am Heart J* 2010;159:612-619.

(21) Completed safety review of Xenical/Alli (orlistat) and severe liver injury. *FDA Drug Safety Communication* [serial online] 2010; Accessed September 15, 2011.

(22) Padwal RS, Majumdar SR. Drug treatments for obesity: orlistat, sibutramine, and rimonabant. *Lancet* 2007;369:71-77.
